# Cost-Effectiveness and Evidence Gaps Surrounding PSMA-PET for Recurrent Prostate Cancer Evaluation

**DOI:** 10.1001/jamanetworkopen.2025.39250

**Published:** 2025-10-24

**Authors:** Natalia Kunst, Jessica B. Long, Preston C. Sprenkle, Isaac Y. Kim, Lawrence Saperstein, Maximilian Rabil, Umar Ghaffar, R. Jeffrey Karnes, Xiaomei Ma, Cary P. Gross, Shi-Yi Wang, Michael S. Leapman

**Affiliations:** 1 Centre for Health Economics, University of York, York, United Kingdom; 2 Cancer Outcomes, Public Policy, and Effectiveness Research Center, Yale School, of Medicine, New Haven, Connecticut; 3 Department of Medicine, Yale School of Medicine, New Haven, Connecticut; 4 Department of Urology, Yale School of Medicine, New Haven, Connecticut; 5 Department of Radiology and Biomedical Imaging, Yale School of Medicine, New Haven, Connecticut; 6 Department of Urology, Mayo Clinic, Rochester, Minnesota; 7 Department of Chronic Disease Epidemiology, Yale School of Public Health, New Haven, Connecticut

## Abstract

**Importance:**

Prostate-specific membrane antigen positron emission tomography (PSMA-PET), a form of molecular imaging, increases detection of advanced and metastatic prostate cancer, but its potential to increase treatment costs due to increased detection is poorly defined.

**Objective:**

To estimate lifetime health and cost outcomes and evaluate the cost-effectiveness associated with implementing PSMA-PET in the clinical evaluation of a patient with a biochemical recurrence (BCR) of prostate cancer, with evaluation of sources of uncertainty in the underlying evidence and assessment of the need and focus of additional data collection.

**Design, Setting, and Participants:**

This economic evaluation developed a decision-analytic model consisting of a decision tree and a Markov model informed by published literature and a study of patients with BCR prostate cancer undergoing PSMA-PET after definitive surgery or radiation therapy at 2 high-volume US academic centers. The analysis was performed between August 1, 2024, and May 1, 2025.

**Interventions:**

Patients underwent (1) PSMA-PET, (2) PSMA-PET imaging as a reflex test if computed tomography plus bone scan (CTBS) was negative or equivocal (CTBS + PSMA-PET), and (3) CTBS alone.

**Main Outcomes and Measures:**

Lifetime quality-adjusted life-years (QALYs) and costs, incremental cost-effectiveness ratio (ICER), and value of information estimates were assessed, with 95% uncertainty intervals (UIs). We assumed a willingness-to-pay threshold of $150 000 per QALY.

**Results:**

The model simulated 1000 patients with BCR (median age, 66 years) and estimated that up-front PSMA-PET is expected to have the highest mean QALYs (7.12 QALYs [95% UI, 6.71-7.51 QALYs]) compared with 6.55 QALYs (95% UI, 6.08-7.03 QALYs) for CTBS; it was also estimated to have higher mean costs ($451 000 [95% UI, $336 000-$577 000]) compared with $351 000 (95% UI, $263 000-$455 000]) for CTBS; consequently, PSMA-PET had mean increments of $99 000 (95% UI, $55 000-153,000) in costs and 0.58 QALYs (95% UI, 0.35-0.82 QALYs) compared with CTBS, leading to an ICER of $172 000 per QALY, which exceeded the assumed $150 000/QALY willingness-to-pay threshold. In patients with lower prostate-specific antigen levels (<2 ng/mL), PSMA had mean increments of $61 000 (95% UI, $21 000-$104 000) in costs and 0.54 QALYs (95% UI, 0.34-0.77 QALYs) compared with CTBS, leading to an ICER of $113 000/QALY, suggesting it had the potential to be cost-effective. Because of high decisional uncertainty, additional information regarding outcomes and diagnostic characteristics may be associated with reduced uncertainty and a gain of 15 747 QALYs.

**Conclusions and Relevance:**

This study found that PSMA-PET may be associated with improved disease detection but may not be cost-effective due to high treatment costs offsetting moderate improvements in QALYs. Further research is needed to identify patient subsets and clinical scenarios in which molecular imaging provides the greatest value.

## Key Points

**Question:**

Is the use of prostate-specific membrane antigen (PSMA) positron emission tomography (PET) for the evaluation of biochemical recurrent (BCR) prostate cancer cost-effective in the US?

**Findings:**

This economic evaluation of modeled patients with prostate cancer found that PSMA-PET had an incremental cost-effectiveness ratio below the willingness-to-pay threshold in patients with BCR and lower prostate-specific antigen levels. However, the decision-analytic model indicated high decision uncertainty and identified that additional data on diagnostic characteristics may be associated with reduction of this uncertainty.

**Meaning:**

This study found that PSMA-PET has the potential to be cost-effective for selected patients with recurrent prostate cancer, but additional evidence is needed to reduce uncertainty about its value across clinical scenarios.

## Teaser

This economic evaluation estimates health and cost outcomes and cost-effectiveness associated with use of prostate-specific membrane antigen positron emission tomography (PSMA-PET) for evaluating biochemical recurrent prostate cancer.

## Introduction

Increasing detection rates, new treatments, and an aging population living longer with advanced prostate cancer continue to drive increases in health care expenditures.^1^ Costs are likely to increase given that many patients who experience biochemical recurrence (BCR) after initial definitive surgery or radiation will undergo additional treatments, including local salvage therapies, and a host of novel systemic agents, including androgen receptor pathway inhibitors, radioligand therapies, and metastasis-directed therapy.^2,3^ Positron emission tomography (PET) imaging targeting prostate-specific membrane antigen (PSMA) enhances the detection and local staging of metastatic prostate cancer compared with conventional computed tomography (CT) and bone scintigraphy (bone scan; CTBS)^4,5^ and has been broadly approved and recommended, including in the US.

Increasing use of PSMA-PET for the evaluation of prostate cancer is expected to alter trajectories of disease management in most patients with BCR.^6^ Assuming modest benefits associated with earlier treatment of metastatic disease, simulation studies comparing diagnostic imaging strategies suggest that the PSMA-PET strategy will be associated with increased quality-adjusted life-years (QALYs) compared with conventional imaging.^6^ However, the evidence underlying long-term clinical outcomes associated with PSMA-PET vs conventional imaging approaches is limited, particularly regarding outcomes associated with earlier identification of metastatic disease. The treatment paradigm for advanced prostate cancer continues to evolve, with the integration of new agents, potentially obscuring the magnitude of differences that are attributable to enhanced detection alone. Despite these uncertainties, empirical evidence suggests that PSMA-PET imaging is commonly associated with earlier use of expensive systemic therapies that are now recommended in the first-line setting.^7^ Tailoring strategies based on prostate-specific antigen (PSA) level, patient preference, and quality and expectancy of life may better balance trade-offs associated with next-generation imaging and requires exploration to mitigate risks of over- or under-detection.^4,5,8,9,10^

In the absence of high-level evidence addressing clinical outcomes associated with the use of PSMA-PET in clinical practice, we applied decision-analytic modeling to estimate lifetime health and cost outcomes and cost-effectiveness associated with imaging strategies for BCR. In addition, we considered varying pretest scenarios based on PSA level at the time of imaging given that this parameter is known to be associated with disease detection and decision-making. Finally, we assessed the underlying evidence, identified drivers of uncertainty in the decision to widely integrate PSMA-PET for the evaluation of BCR in the US, and evaluated the potential need and focus of additional research to resolve decisional uncertainty. This approach aimed to identify the imaging strategy that would be associated with maximum patient health benefits at acceptable resource use and costs.

## Methods

The Yale University Institutional Review Board determined that this economic evaluation was not human participant research and so waived requirements for review and informed consent. Reporting was conducted in accordance with the Consolidated Health Economic Evaluation Reporting Standards Value of Information (CHEERS-VOI) reporting guideline.

### Population

The population of interest included patients with an established diagnosis of prostate cancer who had received and recovered from initial definitive local treatment (radical prostatectomy or radiation therapy) and who had developed BCR, defined as a persistent or an increasing PSA level of 0.20 ng/mL after prostatectomy or a level of 2.0 ng/mL or greater after radiation therapy (to convert to micrograms per liter, multiply by 1). To capture the diversity of clinical scenarios seen in practice, we constructed a pooled model representing the full spectrum of patients with BCR rather than stratifying by prior treatment modality.

### Decision-Analytic Model

**Figure 1.**
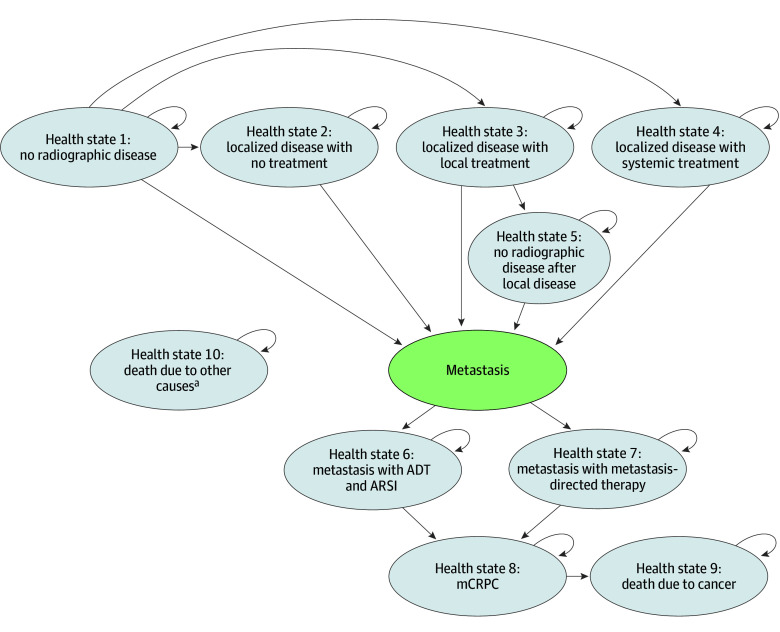
Structure of the Markov Model The model demonstrates mutually exclusive health states that patients may enter after diagnosis of biochemical recurrent prostate cancer. Given differing diagnostic parameters, the probability of entering health states depends on the imaging strategy. Individuals with no radiographic disease (health state 1) are at risk of developing localized (health state 2-4) or metastatic (health state 6-7) disease. Individuals with localized disease (health state 2-4) are at risk of developing metastatic disease. Among individuals with localized disease, only those with local treatment (health state 3) may move to no radiographic disease after localized disease (health state 5). Once metastatic disease has been identified (health state 6-7), patients cannot transition back to localized disease (health state 2-4) or no radiographic disease (health state 1 or 5). Individuals with metastatic disease (health state 6-7) are at risk of progression to metastatic castration-resistant prostate cancer (mCRPC; health state 8). Only individuals who are in the mCRPC state (health state 8) can die from prostate cancer (health state 9). Background mortality (health state 10) is applied to all health states and contributes to death from any cause. ADT indicates androgen deprivation therapy; ARSI, androgen receptor signaling inhibitor. Figure originally published in Kunst et al,^6^ 2024. ^a^Participants in all included health states may encounter background mortality due to death from noncancer causes (health state 10).

We developed a decision-analytic model consisting of a decision tree and a Markov model to simulate long-term health and economic outcomes associated with integrating PSMA-PET imaging into the diagnostic paradigm for the population of interest. The model, which is described in more detail in Kunst et al,^6^ simulates 3 alternative diagnostic imaging strategies: (1) up-front PSMA-PET without conventional imaging, (2) PSMA-PET imaging as a reflex test if CTBS (ie, conventional imaging) is negative or equivocal (CTBS + PSMA-PET), and 3) CTBS without PSMA-PET (CTBS). PSMA-PET was considered the criterion standard imaging modality with the highest diagnostic accuracy. Despite increasing use of PSMA-PET alone, strategy 2 was included to evaluate incremental outcomes associated with up-front conventional imaging, as is still required by some payers.^6^ The decision tree simulated the diagnostic accuracy of each strategy and distributed patients to specific health states in the Markov model (Figure 1). The model assigned probability distributions across a heterogeneous posttreatment population, including individuals with prior prostatectomy, radiotherapy, or other treatments, allowing for subdistributions based on imaging findings and subsequent treatment choices. A detailed description of the decision tree and Markov model is provided in eAppendix 1 in Supplement 1.

In CTBS plus PSMA-PET (strategy 2) and CTBS (strategy 3), we assumed that patients with false-positive results would receive the same treatment or monitoring as those with true-positive findings. For strategy 3, we also assumed that patients with false-negative results, whose true disease state was asymptomatic metastatic prostate cancer, would be treated similarly to those with true-negative results. For PSMA-PET (strategy 1) and CTBS plus PSMA-PET (strategy 2), we assumed that early detection with PSMA-PET had a favorable association with disease progression (transitions between local disease and metastasis and between metastasis and metastatic castration-resistant prostate cancer) as expressed through a hazard ratio for progression with early (diagnosed with PSMA-PET) vs delayed (conventional imaging [CTBS]) treatment of metastasis.^11^ For individuals with false-negative tests detected through CTBS (strategy 3), we assumed increased risks of disease progression. Finally, we assumed that adding metastasis-directed therapy to treatment of metastatic disease would be associated with a reduced hazard of disease progression.^12^

### Input Parameters

The model includes parameters informed with a wide range of data sources (Table 1; eTable in Supplement 1).^11,13,14,15,16,17,18,19,20,21,22,23,24^ These include a retrospective study of patients undergoing PSMA-PET imaging at 2 high-volume academic centers to inform disease characteristics (ie, the proportion of individuals with the disease entering the model and proportion of individuals with localized disease and metastatic disease) and distribution of various treatments.^14^ Cryotherapy was included to reflect its use as salvage local therapy in patients with BCR after initial radiotherapy. The model incorporated a heterogeneous posttreatment population with BCR, and treatment pathways were not stratified by prior definitive therapy. Furthermore, we used published literature to inform CTBS characteristics, disease progression, cancer-related death risk, utility values, and treatment costs.^8,9,11,12,15,17,18,19,20,21,22,23,24,25,26^ All cost inputs were expressed in 2023 US dollars. We used a base estimate of a 44% reduction in the hazard of disease progression associated with earlier detection and treatment.^11^

**Table 1.** Input Parameters for Decision Model

Parameter	Point estimate (95% UI)	Distribution	VOI group^a^	Source
**Demographic characteristics**				
Age, median, y	66	NA	NA	NA
Discount rate, %	3.0	NA	NA	Sanders et al,^13^ 2016
**Disease and diagnostics characteristics**				
Probability of detecting disease with PSMA-PET imaging, %				
Base case	75.12 (71.05-78.80)	β	NA	Yale and Mayo Clinic study; Rabil et al,^14^ 2025
PSA level, ng/mL				
0-1.99	60.19 (54.31-66.52)	β	2	Yale and Mayo Clinic study; Rabil et al,^14^ 2025
2.00-4.99	86.70 (78.07-92.95)	β	NA	Yale and Mayo Clinic study; Rabil et al,^14^ 2025
≥5.00	93.69 89.05-97.14)	β	NA	Yale and Mayo Clinic study; Rabil et al,^14^ 2025
Probability of no radiographic disease with PSMA-PET imaging, %				
Base case				
PSA level, ng/mL	24.91 (21.23-29.07)	β	NA	Yale and Mayo Clinic study; Rabil et al,^14^ 2025
0-1.99	39.75 (33.77-46.24)	β	NA	Yale and Mayo Clinic study; Rabil et al,^14^ 2025
2.00-4.99	13.39 (6.99-21.35)	β	NA	Yale and Mayo Clinic study; Rabil et al,^14^ 2025
≥5.00	6.43 (2.75-10.76)	β	NA	Yale and Mayo Clinic study; Rabil et al,^14^ 2025
Probability of localized disease among patients with detected disease on PSMA-PET imaging, %				
Base case	18.57 (14.77-22.52)	β	NA	Yale and Mayo Clinic study; Rabil et al,^14^ 2025
PSA level, ng/mL				
0-1.99	25.45 (18.71-32.24)	β	2	Yale and Mayo Clinic study; Rabil et al,^14^ 2025
2.00-4.99	14.04 (7.29-22.96)	β	NA	Yale and Mayo Clinic study; Rabil et al,^14^ 2025
≥5.00	13.69 (8.52-19.60)	β	NA	Yale and Mayo Clinic study; Rabil et al,^14^ 2025
Probability of metastatic disease among patients with detected disease on PSMA-PET imaging, %				
Base case	81.46 (77.31-85.38)	β	NA	Yale and Mayo Clinic study; Rabil et al,^14^ 2025
PSA level, ng/mL				
0-1.99	74.49 (67.07-80.88)	β	NA	Yale and Mayo Clinic study; Rabil et al,^14^ 2025
2.00-4.99	86.06 (77.33-92.99)	β	NA	Yale and Mayo Clinic study; Rabil et al,^14^ 2025
≥5.00	86.32 (80.29-91.87)	β	NA	Yale and Mayo Clinic study; Rabil et al,^14^ 2025
Proportion of individuals with LD, conditional on disease detected by CTBS, %	3.42 (0.71-8.32)	β	1	Kane et al,^15^ 2003
Probability of developing metastasis when in health state no radiographic disease after local disease, %	2.27 (1.87-2.71)	β	2	Stish et al,^16^ 2016
HR for progression with early (diagnosed with PSMA-PET) vs delayed (conventional imaging) treatment of metastasis	0.56 (0.49-0.92)	Log normal	1	Meijer et al,^11^ 2022
HR for quicker progression for individuals who were false negative in CTBS	1.79 (1.09-2.05)	Log normal	1	Meijer et al,^11^ 2022
Sensitivity of CTBS, %	37.98 (24.90-52.85)	β	1	Hofman et al,^17^ 2020
Specificity of CTBS, %	91.01 (84.06-96.01)	β	1	Hofman et al,^17^ 2020
Imaging and treatment costs, $				
PSMA-PET	1898 (1242-2727	γ	3	NA
CTBS	1327 (851-1863)	γ	3	NA
Prostatectomy	12 442 (7828-18 163)	γ	NA	Subramanian et al,^18^ 2023
Radiation^b^	17 606 (11 473-25 534)	γ	NA	Parikh et al,^19^ 2020
Cryotherapy	15 845 (9978-22 035)	γ	NA	Subramanian et al,^18^ 2023
ADT	4049 (2548-5769)	γ	3	Ramamurthy et al,^20^ 2019
ARSI	88 492 (56 523-126 237)	γ	3	Ramamurthy et al,^20^ 2019
Metastasis-directed therapy	9096 (5831-13 166)	γ	3	Parikh et al,^19^ 2020
Docetaxel	26 450 (17.570- 38179)	γ	3	Ramamurthy et al,^20^2019
Utility values				
No radiographic disease	0.90 (0.84-0.96)	β	4	Torvinen et al,^21^2013
No radiographic disease after localized disease	0.90 (0.84-0.96)	β	NA	Torvinen et al,^21^2013
Localized disease with no treatment	0.90 (0.84-0.96)	β	NA	Torvinen et al,^21^2013
Localized disease treated with local treatment	0.83 (0.81-0.88)	β	4	Stewart et al,^22^ 2005
Localized disease treated with systemic treatment	0.83 (0.78-0.98)	β	4	Stewart et al,^22^ 2005
Metastatic disease treated with ADT + ARSI	0.8 (0.76-0.84)	β	4	Chi et al,^23^ 2018
Metastatic disease treated with metastasis-directed treatment	0.72 (0.69-0.75)	β	4	Stewart et al,^22^ 2005
mCRPC	0.63 (0.58-0.67)	β	4	Lloyd et al,^24^ 2015

Abbreviations: ADT, androgen deprivation therapy; ARSI, androgen receptor signaling inhibitor; CTBS, computed tomography plus bone scan; HR, hazard ratio; LD, local disease; mCRPC, metastatic castration-resistant prostate cancer; NA, not applicable; PSA, prostate-specific antigen; PSMA-PET, prostate-specific membrane antigen positron emission tomography; UI, uncertainty interval; VOI, value of information.

SI conversion factor: To convert PSA to micrograms per liter, multiply by 1.

^a^ VOI analysis included 4 groups of input parameters: disease characteristics, diagnostic characteristics, imaging and treatment costs, and utility values.

^b^ Cost reflects salvage radiotherapy after prostatectomy using conventional fractionation.

### Statistical Analysis

Applying this model, we simulated lifetime QALYs and costs for all strategies considered. We assessed cost-effectiveness by estimating incremental cost-effectiveness ratios (ICERs), which were estimated after ranking the 3 strategies by their costs. ICER represents incremental costs per QALY gained relative to the next least-costly strategy. We classified a strategy as strongly dominated if it was associated with fewer QALYs at higher costs than the alternative. Strategies that had higher ICERs than more effective strategies were classified as weakly dominated. Dominated and weakly dominated strategies were removed from further consideration. We classified a strategy as cost-effective if it was associated with the highest ICER below the willingness-to-pay threshold considered. In our evaluation, we used the willingness-to-pay threshold of $150 000/QALY and a 3% discount rate, in line with recommendations.^13,27,28^ All analyses were conducted using R statistical software version 4.1.3 (R Project for Statistical Computing) between August 1, 2024, and May 1, 2025.

#### Stratified Analysis

While the base-case analysis examined the 3 strategies for a population with a median PSA level of 1.8 ng/mL, we also performed cost-effectiveness analyses stratified by PSA level at the time of imaging (<2.00, 2.00-4.99, and >5.00 ng/mL). Because of scarce evidence on PSA-specific disease progression, we stratified input parameters based on test results and treatment characteristics from our retrospective study and kept remaining input parameters as in the base-case analysis.

#### Uncertainty Analysis

We propagated uncertainty from model inputs to outcomes of the model with a probabilistic analysis. For this purpose, we first informed all input parameters with appropriate probability distribution. Specifically, we assigned a β distribution to probability parameters and utility weight parameters, a γ distribution to cost parameters, and a log normal distribution to hazard ratios. Probability distributions were informed with precision estimates. For cost parameters, we assumed a 20% uncertainty due to the lack of information about the uncertainty in point estimates. We thereafter performed a Monte Carlo simulation with 10 000 iterations.

The available evidence regarding the long-term clinical outcomes associated with PSMA-PET is based on retrospective studies,^29^ surrogate end points (eg, disease detection and treatment-free survival),^30,31^ and expert opinions, without a mature randomized clinical trial directly examining overall or cancer-related survival. Given uncertainty regarding the benefit associated with earlier disease detection afforded by PSMA-PET, including the potential for treatment-related harms in asymptomatic patients with BCR, we performed a value of information (VOI) analysis to assess the potential benefit associated with obtaining additional evidence surrounding PSMA-PET. This decision-analytic method evaluated how uncertainties in the available evidence were associated with decision-making, assessed outcomes associated with making the decision based on the currently available evidence, and examined the extent to which performing additional research to mitigate these uncertainties may be associated with improved decision-making and increased chances of choosing the intervention that is expected to maximize health benefits at acceptable costs.^32^ Our VOI analysis estimated the maximum potential benefit from collecting additional evidence through expected value of perfect information analysis, an approach that quantifies the difference in expected outcome between perfect and currently available information for all parameters. Thereafter, we identified drivers of uncertainty in the decision about PSMA-PET implementation by applying the expected value of partial perfect information, focusing on individual parameters.^33^ Given that these drivers could represent targets of future research, we grouped relevant input parameters into 4 groups: disease characteristics, diagnostics, utility values, and costs. Specific input parameters included in each group are listed in Table 1 and described in eAppendix 2 in Supplement 1. This grouping was performed based on a relevant research study design that would be most appropriate to collect additional data. Diagnostic, disease characteristic, imaging and treatment cost, and utility value groups are . Given that there are no clear recommendations for willingness-to-pay threshold in the US, our VOI analysis assumed a willingness-to-pay threshold at which the decision uncertainty was the highest (ie, at the peak of VOI) and a discount rate of 3%.^13^ We extrapolated results to the population level assuming a 10-year time horizon and a prevalence and incidence of 50 000 and 40 000 patients with BCR, respectively.

## Results

The model simulated 1000 patients with BCR (median age, 66 years) and estimated that use of up-front PSMA-PET would be expected to have a mean of 7.12 QALYs (95% uncertainty interval [UI], 6.71-7.51 QALYs) compared with an expected mean of 6.55 QALYs (95% UI, 6.08-7.03 QALYs) with CTBS imaging. CTBS plus PSMA-PET would be associated with a slight decrease to an expected mean of 7.03 QALYs (95% UI, 6.63-7.41 QALYs) compared with the PSMA-PET strategy. Furthermore, simulation of the resource use associated with each strategy considered over the lifetime horizon estimated expected mean costs of $451 000 (95% UI, $335 000-$577 000) for up-front PSMA-PET, $459 000 (95% UI, $341 000-$588 000) for CTBS plus PSMA-PET, and $351 000 (95% UI, $263 000-$455 000) for CTBS. Given our assumed willingness-to-pay threshold of $150 000/QALY, this analysis indicated that PSMA-PET would not be considered a cost-effective strategy, with mean increments of $99 000 (95% UI, $55 000-$153,000) in costs and 0.58 QALYs (95% UI, 0.35-0.82 QALYs) incremental QALYs compared with CTBS, for an ICER of $172 000 per QALY gained. CTBS plus PSMA had higher costs and lower health outcomes (ie, was a dominated strategy) and was removed from cost-effectiveness consideration (Table 2).

**Table 2.** Cost-Effectiveness Results for All Patients and by PSA Level

Strategy	Discounted estimate, mean (95% UI)		Incremental estimate, mean (95% UI)		ICER (additional $/QALY gained)^a^
	Cost, $	QALYs	Cost, $	QALYs	
**Base case**					
CTBS	351 000 (263 000 to 455 000)	6.55 (6.08 to 7.03)	NA	NA	NA
PSMA-PET	451 000 (336 000 to 577 000)	7.12 (6.71 to 7.51)	99 000 (55 000 to 153 000)	0.58 (0.35 to 0.82)	172 000
CTBS + PSMA-PET	459 000 (341 000 to 588 000)	7.03 (6.63 to 7.41)	8000 (3000 to 14 000)	−0.09 (−0.04 to −0.18)	Dominated
**PSA level, 0-1.99 ng/mL**					
CTBS	351 000 (265 000 to 455 000)	7.43 (6.84 to 7.99)	NA	NA	NA
PSMA-PET	412 000 (311 000 to 525 000)	7.97 (7.45 to 8.49)	61 000 (21 000 to 104 000)	0.54 (0.34 to 0.77)	113 000
CTBS + PSMA-PET	423 000 (321 000 to 537 000)	7.84 (7.32 to 8.36)	12 000 (5000 to 21 000)	−0.13 (−0.06 to −0.24)	Dominated
**PSA level, 2.00-4.99 ng/mL**					
CTBS	377 000 (279 000 to 492 000)	6.00 (5.33 to 6.72)	NA	NA	NA
PSMA-PET	514 000 (381 000 to 678 000)	6.60 (6.03 to 7.23)	137 000 (77 000 to 207 000)	0.60 (0.36 to 0.84)	230 000
CTBS + PSMA-PET	519 000 (383 000 to 685 000)	6.55 (5.98 to 7.19)	5000 (2000 to 10 000)	−0.05 (−0.02 to −0.11)	Dominated
**PSA level >5.00 ng/mL**					
CTBS	353 000 (256 000 to 458 000)	5.40 (4.89 to 5.93)	NA	NA	NA
PSMA-PET	495 000 (363 000 to 644 000)	6.01 (5.56 to 6.49)	142 000 (82 000 to 214 000)	0.62 (0.38 to 0.88)	230 000
CTBS + PSMA-PET	497 000 (364 000 to 647 000)	5.99 (5.54 to 6.46)	2000 (1000 to 5000)	−0.03 (−0.06 to −0.01)	Dominated

Abbreviations: CTBS, computed tomography plus bone scan; ICER, incremental cost-effectiveness ratio; NA, not applicable; PSMA-PET, prostate-specific membrane antigen positron emission tomography; QALY, quality-adjusted life-year; UI, uncertainty interval.

SI conversion factor: To convert PSA to micrograms per liter, multiply by 1.

^a^ Cost-effectiveness results were expressed in discounted lifetime costs and QALYs associated with each imaging strategy considered. Cost-effectiveness of each strategy was measured using ICERs. An ICER represents incremental costs per QALY gained relative to the next least-costly strategy. An imaging strategy was classified as dominated if it was associated with fewer QALYs at higher costs than the alternative. An imaging strategy was considered cost-effective if it was associated with the highest ICER below the willingness-to-pay threshold considered.

We further assessed cost-effectiveness of the 3 imaging strategies stratified by PSA level at the time of imaging. These results indicated that PSMA-PET was associated with the highest value for money when provided to patients with the lowest PSA levels, of less than 2 ng/mL. For this subpopulation, assuming a $150 000/QALY willingness-to-pay threshold, PSMA-PET would be considered cost-effective given that it had mean increments of $61 000 (95% UI, $21 000-$104 000) in costs and 0.54 QALYs (95% UI, 0.34-0.77 QALYs) compared with CTBS, for an ICER of $113 000/QALY. CTBS plus PSMA-PET remained a dominated strategy (ie, decreased health outcomes at increased costs). For patients with PSA levels greater than 2 ng/mL, our analysis indicated that CTBS may represent the cost-effective strategy given that the ICER of PSMA-PET exceeded the assumed willingness-to-pay threshold (Table 2).

**Figure 2.**
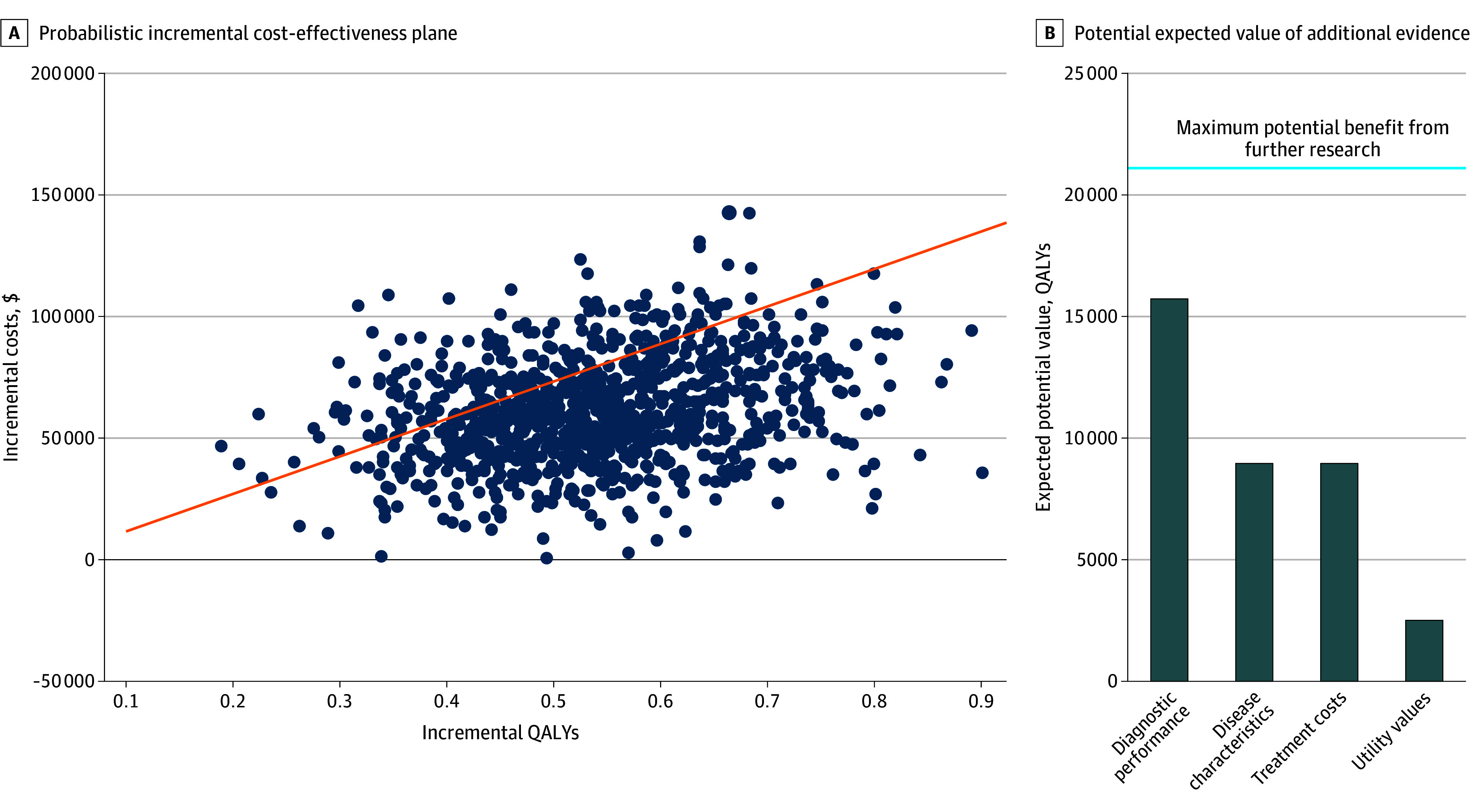
Decision Uncertainty in Positron Emission Tomography Implementation The figure shows uncertainty in the decision on implementing prostate-specific membrane antigen positron emission tomography into the US clinical practice. A, Probabilistic analysis results indicating the number of times results fell under the willingness-to-pay threshold of $150 000 per quality-adjusted life-year (QALY; orange line). B, Potential expected value of additional evidence to improve decision-making on the implementation of prostate-specific membrane antigen positron emission tomography in clinical practice at a willingness-to-pay threshold of $115 000/QALY. Diagnostic, disease characteristic, imaging and treatment cost, and utility value groups are described in eAppendix 2 in Supplement 1.

Probabilistic analysis indicated a 32.0% probability that PSMA-PET would be a cost-effective strategy when provided to all BCR patients (ie, all PSA levels) at a $150 000/QALY willingness-to-pay threshold. For patients with the lowest PSA levels of less than 2 ng/mL, we found a 81.7% probability of cost-effectiveness at a $150 000/QALY willingness to pay threshold and a 52.6% probability at a $115 000/QALY threshold. VOI analysis indicated that the potential negative outcomes associated with making the decision on the use of PSMA-PET in this population were high due to the current uncertainty (Figure 2). Resolving this decision uncertainty could be associated with a gain of 20 869 QALYs at a US population level at the $115 000/QALY willingness-to-pay threshold, at which the decision uncertainty was the highest. Collecting additional data on diagnostic characteristics of imaging strategies considered, which were identified as the main uncertainty driver, could be associated with reduced decision uncertainty and a gain of up to 15 747 QALYs. Other research studies that may be associated with efficient reductions in current uncertainty in the decision on implementation of PSMA-PET in patients with low PSA levels would collect data on disease characteristics, with an associated gain of 8952 QALYs and costs for a gain of 8925 QALYs at a $115 000/QALY willingness-to-pay threshold.

## Discussion

To address the evidence gap regarding long-term health and economic outcomes associated with integrating molecular imaging into the paradigm for recurrent prostate cancer, this economic evaluation developed a decision-analytic model to estimate lifetime outcomes associated with PSMA-PET compared with conventional imaging. We found that PSMA-PET was expected to identify substantially more patients with metastatic disease among those with BCR, with associated increases in the initiation of systemic therapy. As a consequence, PSMA-PET was expected to be associated with increased QALYs but also increased costs and would not be cost-effective at the assumed $150 000/QALY willingness-to-pay threshold. In our PSA-stratified analysis, PSMA-PET yielded the highest QALYs. At the lower PSA distribution, PSMA-PET had an ICER of $113 000/QALY and could be considered cost-effective with the assumed willingness-to-pay threshold. PSMA-PET as a reflex test after negative conventional imaging was associated with increased costs at lower health outcomes in all analyses.

Results of this decision-analytic model suggest that improved detection of metastatic prostate cancer through PSMA-PET would be expected to also be associated with increased prostate cancer health care costs. Owing to increased diagnostic sensitivity, our model estimated the additional detection of more than double the number patients found to have radiographic evidence of metastasis with PSMA-PET vs conventional CTBS at lower PSA levels for which imaging is initially undertaken. Estimates in the Medicare population suggest that health care costs attributable to metastatic prostate cancer have exceeded $8 billion and increase substantially with successive lines of therapy. With routine use of intensified androgen axis therapies, including androgen receptor pathway inhibitors, now recommended for first-line management of hormone-sensitive prostate cancer, annual treatment costs are expected to increase and will be incurred over a longer period.^7,34^ Notably, results of this analysis are the first to our knowledge that consider costs in the US health care system, including contemporary patterns of clinical management favoring intensified systemic therapy.

Our model further projected an increase in QALYs associated with PSMA-PET. These differences were attributable to delays in disease progression assuming benefits associated with earlier treatment of metastatic disease and possible curative intervention of individuals with local recurrence. In the absence of level 1 evidence to guide the magnitude of risk reduction attributable to earlier detection, we applied a base estimate of a 44% reduction in the hazard of disease progression, which has been used in previous studies.^6^ However, substantial uncertainty persists for this estimate given that long-term follow-up of patients diagnosed in the PSMA-PET era has not been completed. Moreover, virtually all available evidence regarding oncologic outcomes associated with PSMA-PET has evaluated surrogate end points, including delayed PSA progression, rather than differences in metastatic progression or survival.^35,36^ These considerations frame results of this decision model and the clinical evidence supporting improved outcomes associated with more sensitive forms of imaging in prostate cancer.

Although this model indicated that PSMA-PET may represent a cost-effective strategy for patients with the lower PSA level, decision uncertainty analysis indicated that widespread integration of PSMA-PET may be associated with negative outcomes if the benefit to this class of more sensitive forms of imaging is less than expected when more evidence is made available. Despite the increasing use of PSMA-PET in the setting of BCR, important evidence gaps remain regarding its clinical utility. Specifically, it is unclear whether the more sensitive detection offered by PSMA-PET is ultimately associated with improved quality or length of life or whether it provides sufficient value to justify increased costs from more intensive imaging and potential downstream interventions. To determine whether further research is worthwhile and design studies that would efficiently resolve that uncertainty, further work should focus on estimating an expected value of sample information and expected net benefit of sampling.

### Limitations

This analysis is limited by the heterogeneity of the BCR setting, which includes various primary treatments, PSA kinetics, patient preferences, and competing comorbidities. These factors influence PSMA-PET performance and subsequent management, and that may not be fully captured in a simplified model. Stratifying by primary treatment type (radiotherapy vs radical prostatectomy) could reduce this variability and is an important area for future research. Accordingly, our conclusions about cost-effectiveness should be interpreted within the context of the model’s assumptions and evidence used. These findings should be further evaluated in other datasets, stratified by primary treatment setting, and under alternative modeling conditions to better capture the heterogeneity of BCR.

Our retrospective study inputs primarily drew on patients with lower PSA distributions, a representative sample of those undergoing PSMA-PET but a subset where conventional imaging has poor diagnostic yield. Thus, these estimates cannot necessarily be extrapolated to patients with very high PSA levels (eg, ≥20 ng/mL), where metastasis detection is likely with CTBS and may not meaningfully alter management or outcomes. Additionally, we assumed that PSMA-PET accurately characterizes disease status and did not account for equivocal findings or variations in imaging interpretations. This assumption may overestimate the performance of PSMA-PET, although we do not expect these differences to affect the direction of these results or their interpretation. In our subpopulation analyses, we used PSA-stratified input parameters on diagnostic outcomes from our retrospective study. While we did not explicitly model the role of PSMA-PET in identifying candidates for newer agents, such as radioligand therapy like ^177^Lu-PSMA, this represents an important additional benefit that warrants evaluation in future work. Additionally, this model considered a single imaging event and did not account for sequential imaging that could occur with conventional imaging and PSMA-PET.

## Conclusions

This economic evaluation found that while the use of PSMA-PET for all patients with recurrent prostate cancer should currently not be considered cost-effective, personalizing decision-making about imaging, such as favoring PSMA-PET among patients with lower PSA distributions in whom salvage therapies may be curative, could represent a cost-effective strategy. This analysis also revealed that resolving high degrees of decisional uncertainty about the long-term outcomes associated with PSMA-PET imaging through additional study could be associated with improved population health.

## Notes

Supplement 1. **eAppendix 1.** Decision tree and Markov model **eAppendix 2.** Diagnostic, disease characteristic, imaging and treatment cost, and utility value parameter groups in value of information analysis **eTable.** Input parameters for decision model examining outcomes associated with PSMA-PET and conventional imaging strategies for the evaluation of biochemical recurrent prostate cancer
eReferences.

Supplement 2.
Data Sharing Statement
