# Supplementary material for: Cost-Effectiveness and Evidence Gaps Surrounding PSMA-PET for Recurrent Prostate Cancer Evaluation
Source: JAMA Netw Open. 2025 Oct 24;8(10):e2539250. doi: 10.1001/jamanetworkopen.2025.39250 (PMC12552925; doi:10.1001/jamanetworkopen.2025.39250)
Supplement: Supplement 1. — eAppendix 1. Decision tree and Markov model eAppendix 2. Diagnostic, disease characteristic, imaging and treatment cost, and utility value parameter groups in value of information analysis eTable. Input parameters for decision model examining outcomes associated with PSMA-PET and conventional imaging strategies for the evaluation of biochemical recurrent prostate cancer eReferences. [file jamanetwopen-e2539250-s001.pdf]

Supplemental Online Content

Kunst N, Long JB, Sprenkle PC, et al. Cost-effectiveness and evidence gaps surrounding PSMA-PET for recurrent prostate cancer evaluation. *JAMA Netw Open*. Published online October 24, 2025. doi:10.1001/jamanetworkopen.2025.39250

eAppendix 1. Decision tree and Markov model

eAppendix 2. Diagnostic, disease characteristic, imaging and treatment cost, and utility value parameter groups in value of information analysis

eTable. Input parameters for decision model examining outcomes associated with PSMA-PET and conventional imaging strategies for the evaluation of biochemical recurrent prostate cancer

eReferences.

This supplemental material has been provided by the authors to give readers additional information about their work.

© 2025 Kunst N et al. *JAMA Network Open*.

eAppendix 1. Decision tree and Markov model

Decision tree

A decision tree was developed to simulate the diagnostic accuracy of each strategy. All patients entering the decision tree had recurrent disease based on detectable PSA levels with definitions differing by prior therapy (radiation versus surgery). PSMA-PET was assumed to be the gold standard imaging modality with the highest diagnostic accuracy, in line with the clinical expert opinion. Consequently, PSMA-PET (Strategy 1) only identifies individuals with true positive and true negative disease. For Strategy 2, individuals with negative CTBS undergo PSMA-PET, ensuring correct disease detection. Positive CTBS include both true positive and false positive tests. Strategy 3, involving only CTBS, leads to a proportion of tests being false positive and false negative. The results of the decision tree distribute patients to specific health states in the Markov model.

Markov model

The Markov model was built to simulate the disease progression of the population of interest after imaging. It consists of 10 health states: 1) no radiographic disease (nRD), 2) local disease (LD) with no treatment, 3) LD with local treatment, 4) LD with systemic treatment, 5) nRD after LD, 6) metastasis with ADT and ARSI, 7) metastasis with metastasis-directed treatment, 8) castration-resistant prostate cancer (mCRPC), 9) death due to prostate cancer, and 10) death due to other causes. The decision tree distributes patients into one of the health states with initial treatment (health state 1-4 and 6-7) after initial imaging. Given differing diagnostic parameters, the probability of entering these health states depends on imaging strategy and imaging result. Individuals in nRD are at risk of developing LD or metastasis. Individuals in LD health states are at risk of developing metastasis. Further, among individuals with LD, only those with local treatment may transition to health state nRD post LD. Once metastasis has been identified, patients do not transition back to LD or nRD. Individuals with metastasis are at risk of progressing to mCRPC. Only those who are in mCRPC state can die due to prostate cancer. Background mortality is applied to all health states. For Strategy 3, we extended the model to include two additional health states: LD False Negative and Metastasis False Negative. We assumed that 10% of these individuals would be appropriately detected each of the

© 2025 Kunst N et al. *JAMA Network Open*.

following years (LD False Negative would move to health states 2-4 and Metastasis False Negative would move to health states 6-7), in line with previously published assumption.[1]

© 2025 Kunst N et al. *JAMA Network Open*.

eAppendix 2. Diagnostic, disease characteristic, imaging and treatment cost, and utility value parameter groups in value of information analysis

Diagnostics group includes 1) proportion of individuals with LD, conditional on disease detected by CTBS; 2) sensitivity of CT with bone scan; 3) specificity of CT with bone scan; 4) hazard ratio for progression with early (diagnosed with PSMA-PET) vs delayed (conventional imaging) treatment of metastasis; 5) hazard ratio for quicker progression for individuals who were False Negative in CT + Bone Scan;

Disease characteristics group includes 1) probability of detecting disease with PSMA-PET imaging PSA 0-1.99; 2) Probability of localized disease among those with detected disease on PSMA-PET imaging PSA 0-1.99; 3) probability of localized disease when in no radiographic disease; 4) probability of developing metastasis when in no radiographic disease; 5) probability of transitioning to no radiographic disease among individuals with localized disease treated with local treatment; 6) probability of developing metastasis when in no radiographic disease post local disease.

Imaging and treatment costs group includes costs of 1) PSMA-PET; 2) CTBS; 3) ADT; 4) ARSI; 5) metastasis-directed therapy; 6) docetaxel.

Utility values group includes utility values of the following health states: 1) no radiographic disease; 2) localized disease treated with local treatment; 3) localized disease treated with systemic treatment; 4) metastatic disease treated with ADT+ARSI; 5) metastatic disease treated with MET-directed therapy; 6) mCRPC.

© 2025 Kunst N et al. *JAMA Network Open*.

eTable. Input parameters for decision model examining outcomes associated with PSMA-PET and conventional imaging strategies for the evaluation of biochemical recurrent prostate cancer

	Parameter	Point estimate (95% Uncertainty Interval)	Distribution	Source
1	Median age (years)	66		
2	Discount rate	3%		
3	Probability of detecting disease with PSMA-PET imaging			
	Base-case	75.12% (71.05-78.80%)	Beta	Yale+Mayo Clinic Study
	PSA 0-1.99	60.19% (54.31-66.52%)	Beta	Yale+Mayo Clinic Study
	PSA 2-4.99	86.70% (78.07-92.95%)	Beta	Yale+Mayo Clinic Study
	PSA ≥5	93.69% 89.05-97.14%)	Beta	Yale+Mayo Clinic Study
4	Probability of no radiographic disease with PSMA-PET imaging			
	Base-case	24.91% (21.23-29.07%)	Beta	Yale+Mayo Clinic Study
	PSA 0-1.99	39.75% (33.77-46.24%)	Beta	Yale+Mayo Clinic Study
	PSA 2-4.99	13.39% (6.99-21.35%)	Beta	Yale+Mayo Clinic Study
	PSA ≥5	6.43% (2.75-10.76%)	Beta	Yale+Mayo Clinic Study
5	Probability of localized disease among those with detected disease on PSMA-PET imaging			
	Base-case	18.57% (14.77-22.52%)	Beta	Yale+Mayo Clinic Study
	PSA 0-1.99	25.45% (18.71-32.24%)	Beta	Yale+Mayo Clinic Study
	PSA 2-4.99	14.04% (7.29-22.96%)	Beta	Yale+Mayo Clinic Study
	PSA ≥5	13.69% (8.52-19.60%)	Beta	Yale+Mayo Clinic Study
6	Probability of metastatic disease among those with detected disease on PSMA-PET imaging			
	Base-case	81.46% (77.31-85.38%)	Beta	Yale+Mayo Clinic Study
	PSA 0-1.99	74.49% (67.07-80.88%)	Beta	Yale+Mayo Clinic Study
	PSA 2-4.99	86.06% (77.33-92.99%)	Beta	Yale+Mayo Clinic Study
	PSA ≥5	86.32% (80.29-91.87%)	Beta	Yale+Mayo Clinic Study
	Proportion of individuals with LD,	3.42% (0.71-8.32%)	Beta	[2]

© 2025 Kunst N et al. *JAMA Network Open*.

	conditional on disease detected by CTBS			
**Transition probabilities***				
7	Probability of localized disease when in no radiographic disease	30.28% (19.33-42.65%)	Beta	[3]
8	Probability of developing metastasis when in no radiographic disease	2.55% (0.49-5.90%)	Beta	[4]
9	Probability of transitioning to no radiographic disease among individuals with localized disease treated with local treatment	54.55% (46-63.56%)	Beta	[3]
10	Probability of developing metastasis when in localized disease with no treatment	4.94% (4.20-5.75%)	Beta	[5]
11	Probability of developing metastasis when in localized disease with local treatment	2.72% (2.03-3.43%)	Beta	[6]
12	Probability of developing metastasis when in localized disease with systemic treatment	5.66% (2.20-10.55%)	Beta	[7]
13	Probability of developing metastasis when in no radiographic disease post local disease	2.27% (1.87-2.71%)	Beta	[8]
14	Probability of mCRPC when in metastatic disease treated with ADT+ARSI	16.11% (14.14-18.11%)	Beta	[9]
15	Probability of death due to prostate cancer when in mCRPC**	34.86% (33.68-36.00%)	Beta	[10]

© 2025 Kunst N et al. *JAMA Network Open*.

Other probabilities				
16	Probability of receiving no treatment among individuals with no radiographic disease	54.63% (45.22-63.89%)	Dirichlet	Yale-Mayo Clinic retrospective study
17	Probability of receiving radiation among individuals with no radiographic disease	13.94% (8.14-20.94%)	Dirichlet	Yale-Mayo Clinic retrospective study
18	Probability of receiving systemic treatment among individuals with no radiographic disease	31.43% (22.79-40.23%)	Dirichlet	Yale-Mayo Clinic retrospective study
19	Probability of receiving no treatment among individuals with localized disease	33.66% (22.37-45.46%)	Dirichlet	Yale-Mayo Clinic retrospective study
20	Probability of receiving local treatment among individuals with localized disease	21.04% (11.90-31.76%)	Dirichlet	Yale-Mayo Clinic retrospective study
21	Probability of receiving systemic treatment among individuals with localized disease	45.29% (33.15-58.42%)	Dirichlet	Yale-Mayo Clinic retrospective study
22	Probability of receiving prostatectomy among individuals with localized disease treated with local treatment	2.59% (0.57-6.05%)	Dirichlet	Yale-Mayo Clinic retrospective study
23	Probability of receiving radiation among individuals with localized disease treated with local treatment	85.93% (78.63-91.46%)	Dirichlet	Yale-Mayo Clinic retrospective study
24	Probability of receiving cryotherapy among individuals with localized disease	11.48% (6.40-17.94%)	Dirichlet	Yale-Mayo Clinic retrospective study

© 2025 Kunst N et al. *JAMA Network Open*.

	treated with local treatment			
25	Probability of receiving systemic treatment among individuals with metastatic disease	79.62% (74.72-84.26%)	Beta	Yale-Mayo Clinic retrospective study
26	Probability of receiving metastasis-directed treatment among individuals with metastatic disease	20.38% (15.75-25.28%)	Beta	Yale-Mayo Clinic retrospective study
**Hazard ratios**				
27	Hazard ratio for mCRPC from metastatic disease treated with MET-directed compared to treated with ART+ARSI	0.25 (0.12-0.55)	Lognorm	[11]
28	Hazard ratio for progression with early (diagnosed with PSMA-PET) versus delayed (conventional imaging) treatment of metastasis	0.559 (0.488-0.917)	Lognorm	[12]
29	Hazard ratio for quicker progression for individuals who were False Negative in CT + Bone Scan	1.79 (1.09-2.05)	Lognorm	[12]
**Test accuracy**				
30	Sensitivity of CT with bone scan	38% (24-52%)	Beta	[13]
31	Specificity of CT with bone scan	91% (85-97%)	Beta	[13]
**Imaging and treatment costs**				
	PSMA-PET	1,898 (1,242.32-2,726.85	Gamma	
	CTBS	1,327 (850.47-1,862.65)	Gamma	
32	Prostatectomy	12,442 (7,828.95-18,163.18)	Gamma	[14]
33	Radiation	17,606	Gamma	[15]

		(11,473.25-25,534.75)		
34	Cryotherapy	15,845 (9,978.37-22,035.64)	Gamma	[14]
35	ADT	4,049 (2,548-5,769.93)	Gamma	[16]
36	ARSI	88,492 (56,523.4-126,237.7)	Gamma	[16]
37	Metastasis directed therapy	9,096 (5,831.39-13,166.38)	Gamma	[15]
38	Docetaxel	26,450 (17.570-38179)	Gamma	[16]
**Utilities values**				
39	No radiographic disease	0.90 (0.84-0.96)	Beta	[17]
40	No radiographic disease post localized disease	0.90 (0.84-0.96)	Beta	[17]
41	Localized disease with no treatment	0.90 (0.84-0.96)	Beta	[17]
42	Localized disease treated with local treatment	0.831 (0.805-0.878)	Beta	[18]
43	Localized disease treated with systemic treatment	0.83 (0.78-0.98)	Beta	[18]
44	Metastatic disease treated with ADT+ARSI	0.8 (0.76-0.84)	Beta	[19]
45	Metastatic disease treated with MET-directed treatment	0.72 (0.69-0.75)	Beta	[18]
46	mCRPC	0.625 (0.577-0.673)	Beta	[20]

© 2025 Kunst N et al. *JAMA Network Open*.

eReferences.

1. Kunst N, Long JB, Westvold S, et al. Long-Term Outcomes of Prostate-Specific Membrane Antigen-PET Imaging of Recurrent Prostate Cancer. *JAMA Netw Open*. Oct 1 2024;7(10):e2440591. doi:10.1001/jamanetworkopen.2024.40591
2. Kane CJ, Amling CL, Johnstone PA, et al. Limited value of bone scintigraphy and computed tomography in assessing biochemical failure after radical prostatectomy. *Urology*. Mar 2003;61(3):607-11. doi:10.1016/s0090-4295(02)02411-1
3. Schmidt-Hegemann NS, Stief C, Kim TH, et al. Outcome after PSMA PET/CT based salvage radiotherapy in patients with biochemical recurrence after radical prostatectomy: a bi-institutional retrospective analysis. *J Nucl Med*. Feb 1 2019;60(2):227-233. doi:10.2967/jnumed.118.212563
4. Bianchi L, Ceci F, Costa F, et al. The Impact of PSMA-PET on Oncologic Control in Prostate Cancer Patients Who Experienced PSA Persistence or Recurrence. *Cancers (Basel)*. Dec 30 2022;15(1)doi:10.3390/cancers15010247
5. Pound CR, Partin AW, Eisenberger MA, Chan DW, Pearson JD, Walsh PC. Natural history of progression after PSA elevation following radical prostatectomy. *Jama*. May 5 1999;281(17):1591-7. doi:10.1001/jama.281.17.1591
6. Stephenson AJ, Shariat SF, Zelefsky MJ, et al. Salvage radiotherapy for recurrent prostate cancer after radical prostatectomy. *Jama*. Mar 17 2004;291(11):1325-32. doi:10.1001/jama.291.11.1325
7. Freedland SJ, Humphreys EB, Mangold LA, Eisenberger M, Dorey FJ, Walsh PC, Partin AW. Risk of prostate cancer-specific mortality following biochemical recurrence after radical prostatectomy. *Jama*. Jul 27 2005;294(4):433-9. doi:10.1001/jama.294.4.433
8. Stish BJ, Pisansky TM, Harmsen WS, Davis BJ, Tzou KS, Choo R, Buskirk SJ. Improved Metastasis-Free and Survival Outcomes With Early Salvage Radiotherapy in Men With Detectable Prostate-Specific Antigen After Prostatectomy for Prostate Cancer. *J Clin Oncol*. Nov 10 2016;34(32):3864-3871. doi:10.1200/jco.2016.68.3425
9. Davis ID, Martin AJ, Stockler MR, et al. Enzalutamide with Standard First-Line Therapy in Metastatic Prostate Cancer. *N Engl J Med*. Jul 11 2019;381(2):121-131. doi:10.1056/NEJMoa1903835
10. Shore ND, Laliberté F, Ionescu-Ittu R, et al. Real-World Treatment Patterns and Overall Survival of Patients with Metastatic Castration-Resistant Prostate Cancer in the US Prior to PARP Inhibitors. *Adv Ther*. Aug 2021;38(8):4520-4540. doi:10.1007/s12325-021-01823-6
11. Tang C, Sherry AD, Haymaker C, et al. Addition of Metastasis-Directed Therapy to Intermittent Hormone Therapy for Oligometastatic Prostate Cancer: The EXTEND Phase 2 Randomized Clinical Trial. *JAMA Oncol*. Jun 1 2023;9(6):825-834. doi:10.1001/jamaoncol.2023.0161
12. Meijer D, Eppinga WSC, Mohede RM, et al. Prostate-specific Membrane Antigen Positron Emission Tomography/Computed Tomography Is Associated with Improved Oncological Outcome in Men Treated with Salvage Radiation Therapy for Biochemically Recurrent Prostate Cancer. *Eur Urol Oncol*. Apr 2022;5(2):146-152. doi:10.1016/j.euo.2022.01.001
13. Hofman MS, Lawrentschuk N, Francis RJ, et al. Prostate-specific membrane antigen PET-CT in patients with high-risk prostate cancer before curative-intent surgery or radiotherapy (proPSMA): a prospective, randomised, multicentre study. *Lancet*. Apr 11 2020;395(10231):1208-1216. doi:10.1016/s0140-6736(20)30314-7
14. Subramanian K, Martinez J, Huicochea Castellanos S, et al. Complex implementation factors demonstrated when evaluating cost-effectiveness and monitoring racial disparities associated with [(18)F]DCFPyL PET/CT in prostate cancer men. *Sci Rep*. May 23 2023;13(1):8321. doi:10.1038/s41598-023-35567-w
15. Parikh NR, Chang EM, Nickols NG, et al. Cost-Effectiveness of Metastasis-Directed Therapy in Oligorecurrent Hormone-Sensitive Prostate Cancer. *Int J Radiat Oncol Biol Phys*. Nov 15 2020;108(4):917-926. doi:10.1016/j.ijrobp.2020.06.009
16. Ramamurthy C, Handorf EA, Correa AF, Beck JR, Geynisman DM. Cost-effectiveness of abiraterone versus docetaxel in the treatment of metastatic hormone naïve prostate cancer. *Urol Oncol*. Oct 2019;37(10):688-695. doi:10.1016/j.urolonc.2019.05.017
17. Torvinen S, Färkkilä N, Sintonen H, Saarto T, Roine RP, Taari K. Health-related quality of life in prostate cancer. *Acta Oncol*. Aug 2013;52(6):1094-101. doi:10.3109/0284186x.2012.760848

© 2025 Kunst N et al. *JAMA Network Open*.

18. Stewart ST, Lenert L, Bhatnagar V, Kaplan RM. Utilities for prostate cancer health states in men aged 60 and older. *Med Care*. Apr 2005;43(4):347-55. doi:10.1097/01.mlr.0000156862.33341.45
19. Chi KN, Protheroe A, Rodríguez-Antolín A, et al. Patient-reported outcomes following abiraterone acetate plus prednisone added to androgen deprivation therapy in patients with newly diagnosed metastatic castration-naive prostate cancer (LATITUDE): an international, randomised phase 3 trial. *Lancet Oncol*. Feb 2018;19(2):194-206. doi:10.1016/s1470-2045(17)30911-7
20. Lloyd AJ, Kerr C, Penton J, Knerer G. Health-Related Quality of Life and Health Utilities in Metastatic Castrate-Resistant Prostate Cancer: A Survey Capturing Experiences from a Diverse Sample of UK Patients. *Value Health*. Dec 2015;18(8):1152-7. doi:10.1016/j.jval.2015.08.012

© 2025 Kunst N et al. *JAMA Network Open*.
