# Supplementary material for: Cost-Effectiveness and Evidence Gaps Surrounding PSMA-PET for Recurrent Prostate Cancer Evaluation
Source: JAMA Netw Open. 2025 Oct 24;8(10):e2539250. doi: 10.1001/jamanetworkopen.2025.39250 (PMC12552925; doi:10.1001/jamanetworkopen.2025.39250)
Supplement: Supplement 2. — Data Sharing Statement [file jamanetwopen-e2539250-s002.pdf]

Data Sharing Statement

Kunst. Cost-Effectiveness and Evidence Gaps Surrounding PSMA-PET for Recurrent Prostate Cancer Evaluation. *JAMA Netw Open*. Published online October 24, 2025. doi:10.1001/jamanetworkopen.2025.39250

Data
Data available: No
